# The role of cGMP-cGKI-signaling for duodenal bicarbonate secretion

**DOI:** 10.1186/1471-2210-11-S1-P69

**Published:** 2011-08-01

**Authors:** Beate Spiessberger, Dieter Saur, Ursula Seidler, Anurag K Singh, Robert Lukowski, Franz Hofmann

**Affiliations:** 1 For 923 at Institut für Pharmakologie und Toxikologie, TU München, Germany; 2 Department of Internal Medicine II, Klinikum Rechts der Isar, TU München, Germany; 3 Department of Gastroenterology, Hepatology and Endocrinology, Hannover Medical School, Germany; 4 Institute of Pharmacy, Department of Pharmacology, Toxicology and Clinical Pharmacy, University of Tübingen, Germany

## Background

The duodenal mucosa protects itself from gastric acid injury by the secretion of bicarbonate.

We determined a possible role of the cGMP-cGKI pathway for the duodenal bicarbonate secretion by studying conventional cGKI knockouts (cGKI-KOs) and rescue mice (RM) that express either the cGKIα or β isoform in SM22α positive smooth muscle cells [1].

## Results

Gastric acid production was similar in control and mutant mice. The basal secretion rate of bicarbonate was strongly reduced in the different gene-targeted cGKI mice.

Protons induced bicarbonate secretion in controls but not in mutant mice. The dysfunction of the duodenal bicarbonate secretion of RM and cGKI-KO animals was associated with severe gastrointestinal bleedings, which were caused by the age-dependent aggravation of an epithelial ulceration that localized to the papilla Vateri.

In conclusion, the analysis of cGKI-KO and RM indicates that a cGMP-cGKI-dependent pathway is present in non-smooth muscle cells of the duodenum, that is involved in the basal and acid-induced secretion of bicarbonate. We suppose, that cGKI might be expressed in the enteric nervous system. Furthermore we hypothesize a crucial role for cGKI signaling via the N. vagus.

In contrast to the widespread assumption, that cGKI is expressed in duodenal cells of Cajal we were not able to detect any cGKI in these cells.

## Conclusion

The inability to secrete adequate amounts of bicarbonate ultimately leads to duodenal ulceration. We postulate that the continuous blood loss accounts for the chronic anemia of cGKI mutant mice and causes the premature death of the cGKI-KOs and RM.

To administer a therapy against the ulceration and their effects, cGKI knockout mice were treated with a proton pump inhibitor. Chronical treatment of conventional cGKI knockout mice with the proton pump inhibitor esomeprazol prolonged their life expectancy significantly in contrast to the untreated cGKI-knockout mice.
